# Vulnerable parenting among mothers with substance abuse in their family of origin: a cross-sectional comparative study of mothers in an infant and toddler program

**DOI:** 10.1186/s40064-016-3045-0

**Published:** 2016-09-13

**Authors:** Eva Tedgård, Maria Råstam

**Affiliations:** 1 Department of Clinical Sciences Lund, Child and Adolescent Psychiatry, Lund University, Baravägen 1, 22185 Lund, Sweden; 2 Gillberg Neuropsychiatry Centre, Institute of Neuroscience and Physiology, University of Gothenburg, Göteborg, Sweden; 3 Office for Healthcare ‘Sund’, Child and Adolescent Psychiatry, Infant and Toddler Unit, 20502 Malmö, Sweden; 4 Beritta Gurrisgatan 17, 217 74 Malmö, Sweden

**Keywords:** Parenting, Family of origin, Substance abuse, ADHD, Depression

## Abstract

**Objective:**

To investigate whether women raised in a family with substance abuse constitute a particularly vulnerable group of patients in an infant psychiatry setting and to identify the risk factors of suspected parental malfunctioning in women referred to treatment in an infant and toddler intervention program.

**Background:**

A history of family substance abuse can severely disrupt the caretaking abilities of parents in ways that can have far-reaching consequences, and children growing up with insufficient parental care may incorporate this deficiency into their own parental behavior.

**Methods:**

In total, 126 mothers completed self-report questionnaires assessing their substance abuse and health problems as well as problems in their family of origin. The index group was defined as women who reported substance abuse in their family of origin (n = 35). The comparison group was defined as women who denied substance abuse in their family of origin (n = 91).

**Results:**

Symptoms of depression and anxiety were overrepresented in the total group of mothers compared with the Swedish norm. The index group had experienced parental divorce and traumatic life events more often and reported earlier substance abuse of their own. They had significantly more depression and ADHD symptoms and were more often single parents. All these factors can have a negative influence, separately or in combination, on the ability to practice sensitive parenting.

**Conclusions:**

Female offspring of substance-abusing parents are an especially vulnerable group of patients. To prevent the intergenerational transmission of alcohol and drug abuse, it is important to identify parents with specific needs and to administer targeted treatment and support at primary health care centers and child psychiatric clinics.

## Background

Many factors can have a negative influence on an individual’s parenting abilities. For example, it is well documented that relationships in one’s family of origin are an important factor affecting parenting skills (Solomon and George 2011). Parents who view their own early caregivers as hostile and insensitive are thought to be more likely to provide a more insecure environment for their own children (Liotti 2004; Solomon and George 1999, 2011). Substance abuse in the family of origin is another factor that can negatively affect parenting behavior (Howe 2005; Locke and Newcomb 2004). Parenting capability can also be influenced by a parent’s experiences of an earlier traumatic life event (Cohen et al. 2008), health problems such as depression (Murray et al. 2011; Reck et al. 2008; Tronick and Reck 2009), anxiety (O’Connor et al. 2005), and attention deficit hyperactivity disorder (ADHD) (Chronis-Tuscano et al. 2008; Johnston et al. 2012), and substance abuse (Kroll 2004; Suchman et al. 2008).

The influence of parents’ substance abuse on the health of their offspring is far-reaching (Anda et al. 2002; Bakoyiannis et al. 2014; Balsa et al. 2009; Elkins et al. 2004; Velleman et al. 2008) and can severely disrupt parents’ caretaking abilities. Parental substance use disorder has been associated with increased aggression and vulnerability to stress in the affected offspring (Elkins et al. 2004; Dube et al. 2001). Studies have found associations between substance abuse in parents and the development of anxiety and affective disorder in their children (Chen and Weitzman 2005; Foley et al. 2004; Hill et al. 2008; Kelley et al. 2011); researchers have also found that children of parents who abuse substances are at an increased risk of experiencing substance abuse themselves (Buu et al. 2009; Kendler et al. 2015; Yule et al. 2013). Female offspring of women with alcoholism appear to have a greater risk of adult psychopathologies than male children (Morgan et al. 2010; Pearson et al. 2012). Children of parents with alcohol and/or drug abuse are also at risk of developing ADHD (Knopik et al. 2006), particularly children in families with multiple cases of alcohol abuse (Hill et al. 2008). Numerous studies have demonstrated a considerable overlap between ADHD and substance use disorder (Lee et al. 2011) (Iacono et al. 2008; Young et al. 2009). ADHD has also been found to be a significant predictor of substance abuse (Capusan et al. 2016; Wilens et al. 2011), and at least one in four individuals with substance abuse have ADHD (Ohlmeier et al. 2008; Sullivan and Rudnik-Levin 2001). It has been suggested that the dual diagnosis of substance abuse and ADHD carries an added risk of negative parenting (Johnston et al. 2012).

In the past decade, infant and toddler programs have been developed in child and adolescent psychiatry to help families with parenting problems promote child health and wellbeing and to support sensitive parenting. The most common problems in this setting are regulatory disorders in the child and mental health problems of the mother.

The aim of these intervention programs is to improve the parent–child relationship, increase parental responsiveness and reflectivity and parent–child emotional regulation and establish a secure attachment between the child and his or her parent/s. The intervention has a combination of interaction treatment were the parents are encouraged to become attentive to the child’s emotions and initiatives of contact and psychotherapy for the parent (Neander and Engstrom 2009). The parents who participate in such programs have all exhibited impaired parenting behavior, but as a group, they are heterogeneous. An earlier report from an infant and toddler program in Sweden has found that approximately half of the mothers who joined the program with their infants had been raised with substance-abusing parents. Furthermore, these women, compared with other parents reported more symptoms in their children, more severe problems between them and their children and greater difficulty including the father in the treatment (Tedgård 2008). It is important to identify parents with pronounced needs to provide targeted treatment.

The objective of the present study was to investigate whether women raised in a family with substance abuse constitute a particularly vulnerable group of patients in an infant psychiatry setting and to identify risk factors for suspected parental malfunctioning in women referred to the intervention program for parent–child relationship problems. We compared women with and without substance abuse in their family of origin regarding psychosocial background factors, such as traumatic life events, parental separation, and family history of physical and mental health. We further explored participants’ symptoms of depression, anxiety, ADHD, and substance abuse. Individuals with ADHD experience problems parenting (Knopik et al. 2006), but the frequency of ADHD among individuals seeking treatment for parenting problems has not been investigated to date.

We hypothesized that a woman’s experience of substance abuse in her family of origin would be associated with more symptoms of anxiety, depression and higher risk of own substance abuse, thus putting her at a special risk for malfunctioning parenting.

Second, we hypothesized that there would be an increased prevalence of ADHD in our total sample of individuals with parenting problems and an even greater increased occurrence of symptoms indicative of ADHD in the subsample of individuals who had been raised in a family with substance abuse.

## Methods

The current study focused on self-reported data from women participating with their children (aged 0–4 years) in a parent–child intervention program in a specialized outpatient infant and toddler psychiatric clinic in Malmö, Sweden, because of deficient parenting behavior and dysfunctional patterns of interaction with their child. All participants were referred for treatment because of parental problems either through self-referral (60 %) or through referral from a doctor or nurse from a primary care or pediatric clinic. Parents diagnosed with intellectual disabilities were not treated at the unit. The data were collected before the start of the intervention. The data are stored in a database titled “Factors important for parenting” (PUL), with the Skane Region County Council.

### Subjects

**Fig. 1:**
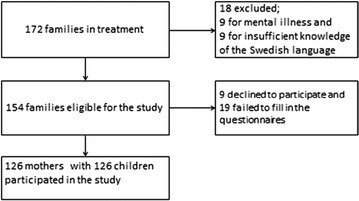
Flow chart of the families in the study

During the study period from May 1, 2011, to May 1, 2013, a total of 172 families were admitted to the clinic (Fig. 1).

The exclusion criteria were severe mental illness (psychosis or severe depression with hospitalization; n = 9) or deficient knowledge of the Swedish language (n = 9), and 153 families were eligible. Nine families declined participation, and a further 19 failed to complete the questionnaires.

In the present study, only data from the mothers were included because the number of participating fathers was too small to conduct statistical analyses. Altogether, 126 mothers participated in the study with their children. In two families, both parents were women, and one in each pair was randomly selected to participate (Fig. 1).

In the next step, the total sample was divided according to substance abuse in the family of origin. The index group was defined as women who reported alcohol and/or drug abuse in their family of origin, and the comparison group (COMP) was defined as women who denied alcohol and/or drug abuse in their family of origin.

### Instruments

*A self*-*reported questionnaire* comprising 13 questions was specifically designed by the research group for the present study; it included questions pertaining to sociodemographic and psychosocial risk factors, such as traumatic life events, and somatic and psychiatric disorders, including substance abuse by the subjects and in the family of origin. The questionnaire included dichotomous questions, and the participants were asked to provide a more detailed answer to any initial positive responses (e.g., ‘did you or any family member in your family of origin suffer from substance abuse problems? (Alcohol, prescribed drugs, illegal drugs) If yes, what kind?’) (Table 1).

**Table 1:**
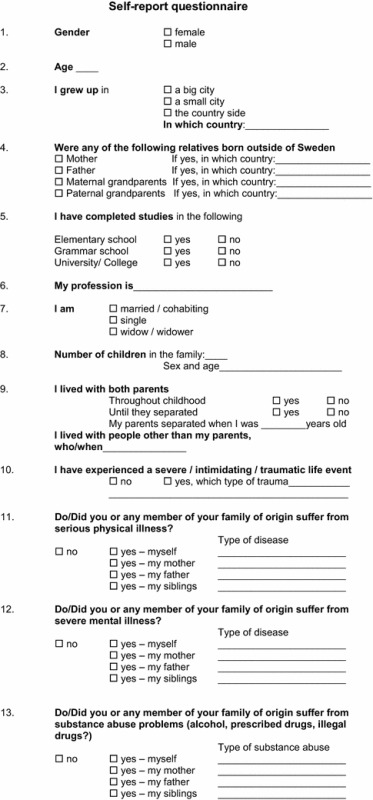
Self-report questionnaire

The *Hospital Anxiety and Depression Scale (HADS)* have been shown to be valid and reliable in medical practice and research (Snaith 2003; Zigmond and Snaith 1983). The HADS is a Likert-style questionnaire consisting of 14 items (seven for anxiety and seven for depression). The score ranges from 0 (no anxiety) to 3 (greatest anxiety) for each question (Zigmond and Snaith 1983). The ranges of scores used to define cases are as follows: 0–7, normal; 8–10, mild disorder; 11–14, moderate disorder and 15–21, and severe disorder (Bowling 2005; McDowell 2006; Snaith 2003). A cutoff score of 8 was used in the present study (Bjelland et al. 2002). The results were compared with the Swedish norm (Lisspers et al. 1997). Previous studies have supported the two-factor model used in this study (Helvik et al. 2011; Norton et al. 2013).

The *Alcohol Use Disorders Identification Test (AUDIT)* is an internationally well-validated questionnaire assessing alcohol use (Saunders et al. 1993). Alcohol consumption was measured by using the Swedish version of the AUDIT. The instrument consists of ten questions, each of which is scored from 0 to 4 points; the maximum score is thus 40. It contains questions pertaining to the level and frequency of alcohol consumption and heavy drinking, drinking behavior, and alcohol-related problems. Harmful use of alcohol is indicated by a score of 6–13 points for women and 8–15 points for men, and alcohol dependence is indicated by a score ≥18 points. The recommended cutoff scores, ≥6 for women and ≥8 for men, were used in this study (Berman et al. 2012).

The *Drug Use Disorders Identification Test (DUDIT)* is a thoroughly validated questionnaire evaluating the use of illegal drugs (Berman et al. 2005). Patients at risk from drug use was determined by using the Swedish version of the DUDIT. The instrument consists of eleven items designed to assess the consumption patterns of illicit drugs and problems related to drug use. The first nine items are scored on a five-point scale ranging from 0 to 4, and the last two are scored on a three-point scale with the values 0, 2 and 4. Thus, the total scores range from 0 to 44, with higher scores suggesting a more severe drug problem. The DUDIT cutoff score for any type of problematic use (i.e., harmful use, substance abuse, or dependence) is generally recommended to be six for men and two for women. The following risk levels are suggested for the DUDIT scores: no drug-related problems (total scores 0–5/1), possible drug-related problems, i.e., risky or harmful drug habits that might be diagnosed as substance abuse/harmful use or dependence (6/2–24), and probable heavy dependence on drugs (scores ≥25).

The *ASRS v. 1.1 Adult ASRS Full Edition (WHO Adult ADHD Self*-*Report Scale*) is the WHO’s self-report rating scale for adult ADHD (Kessler et al. 2005). The scale consists of 18 items that are consistent with the *DSM*-*IV* diagnostic criteria for ADHD symptoms. Items 1–9 (Part A) reflect symptoms of inattention, and items 10–18 (Part B) reflect symptoms of hyperactivity or impulsivity. The internal consistency of the ASRS in this dataset on the basis of Cronbach’s alpha was .86 for Part A and .86 for Part B. ADHD symptoms were assessed by using the Swedish version of the 18-item ASRS. A higher cut-off level of ≥24 was used in this study. All instruments have been validated for the Swedish population.

The Swedish Parenthood Stress Questionnaire (SPSQ) (Ostberg et al. 1997) is based on the Parent Domain of the Parenting Stress Index (Abidin 1995). The SPSQ measures parental stress and comprises 34 items divided into five subscales: incompetence, role restriction, social isolation, spouse relationship problems, and health problems. Together, these five subscales form an overall parenting stress score. The response options range from ‘strongly agree’ to ‘strongly disagree,’ which are scored on a Likert-type scale from 1 to 5. Higher scores indicate more stress. The instrument has been used in several studies and has shown good psychometric properties.

The measures were administered by a well-experienced administrator who had participated in other studies and had a personal contact with each family. At admission, the parents were asked by the administrator to participate in the study. The administrator was then responsible for the distribution and collection of all the instruments.

### Statistical analysis

The data analysis was performed using IBM SPSS 22.0 software for Windows. Descriptive statistics with means, medians and standard deviations were used to describe the findings. In the analysis of the findings, two-sample *t* tests and Pearson’s Chi square test for non-parametric data were used to identify significant differences between the index group and the comparison group. Statistical reasoning was performed on the basis of the statistical tests, with a significance level of .05.

### Ethics

Written informed consent was obtained from all participants. The study was approved by the Regional Ethics Committee at Lund University, Sweden.

## Results

### Sociodemographic data

In 66 % of the 126 families, the mother had a Swedish background, and there were no differences between groups. The participants were between 19 and 46 years of age (mean age 32 years; SD 5.32), and there were no significant differences in age between the index and the COMP group. Sixty-seven percent of the mothers were primiparous, and there were no differences between groups. The 126 children (52 girls and 74 boys) were between 2 and 48 months old; 61 % were younger than 12 months, and 84 % of all mothers were married or cohabitating. Being a single mother was more common in the index group (29 vs. 11 %, *p* = .048). There were no differences in the level of education or employment status (Table 2).

**Table 2:** Sociodemographic and psychosocial characteristics of the sample (n = 126)

	Index women	COMP women	*p* value
	n = 35	n = 91	
Age of mother (years)	30.5 (SD 5.8)	32.5 (SD 4.9)	.075^a^
Primiparous	23 (66 %)	63 (69 %)	.766^b^
Age of child (months)	17.49 (SD 14.0)	13.94 (SD 13.8)	.189^a^
Married/cohabitating	25 (71 %)	81 (89 %)	.048^b^
Educational background			.225^b^
Elementary <9 years	5 (14 %)	5 (6 %)	
Grammar/secondary 10–12 years	14 (40 %)	35 (39 %)	
College/university >12 years	16 (46 %)	52 (56 %)	
Unemployed	6 (17 %)	15 (17 %)	.929^b^
Mother’s ethnicity			.038^b^
Swedish origin	19 (54 %)	64 (70 %)	
Scandinavian origin	5 (14 %)	2 (2 %)	
European origin	6 (17 %)	17 (19 %)	
“Outside Europe” origin	5 (14 %)	8 (9 %)	

^a^ *t* test

^b^Pearson’s Chi square

### Substance abuse in parents in the family of origin

Of the participating women, 35 (27 %) reported that they were raised in families with substance abuse. This group constituted our index group. In this group, 10 women reported that both of their parents abused substances during their childhood, 15 reported that only their father abused substances, and 10 reported that only their mother abused substances. Alcohol abuse was the most common form of abuse. The COMP group consisted of 91 women who denied alcohol and/or drug abuse in their family of origin.

### Mental/physical illness and divorce in the family of origin

There were no significant differences in the reported mental (41 vs. 38 %, *p* = .835) or physical health (29 vs. 35 %, *p* = .570) of the family of origin between groups. Divorce in the family of origin (66 vs. 38 %, *p* = .005) and having a sibling with substance abuse (29 vs. 4 %, *p* = .0001) were significantly more common in the index group (Table 3).

**Table 3:** Physical and psychological illness and divorce in the family of origin

	Index women	COMP women	*p* value
	n = 35	n = 91	
Physical illness in the family of origin	10 (29 %)	32 (35 %)	.570
Psychological illness in the family of origin (except for SUD)	14 (41 %)	35 (38 %)	.835
Divorce in the family of origin	23 (66 %)	35 (38 %)	.005
Sibling with substance abuse	10 (29 %)	4 (4 %)	.0001

Pearson’s Chi square

*SUD* substance use disorder

### Previous substance abuse in participants

The women in the index group self-reported previous personal substance abuse significantly more often than women in the COMP group (20 vs. 1 %, *p* = .0001).

### Traumatic life events in participants

The majority of the women reported traumatic life events (64 %). The most commonly reported events were severe physical abuse (n = 30), sexual abuse (n = 14), death in the immediate family (n = 10), accidents (n = 2), and having witnessed violence (n = 7). Traumatic life events were more common in the index group than in the COMP group (84 vs. 56 %, *p* = .004).

### Self-reported psychiatric symptoms at admission

#### Anxiety symptoms according to the HADS

Of the 126 women in the sample, 104 (82 %) had anxiety symptom scores that were above the cut-off (≥8), and there were no significant differences between the index and COMP groups (83 vs. 82 %, *p* = .225; Table 4).

**Table 4:** Self-report questionnaires, index women compared with COMP women

	Index women	COMP women	*p* value
	n = 35	n = 91	
HADS Symptoms of anxiety	29 (83 %)	75 (82 %)	.225
HADS Symptoms of depression	19 (54 %)	33 (36 %)	.032
AUDIT Harmful use of alcohol (past 12 months)	5 (14 %)	6 (7 %)	.171
DUDIT Drug-related problems (past 12 months)	6 (18 %)	5 (6 %)	.033
ASRS Highly probable ADHD	11 (31 %)	13 (14 %)	.019

Pearson’s Chi square

#### Depression symptoms according to HADS

Of the 126 women in the sample, 52 (41 %) reported depression symptom scores above the cut-off (≥8). Symptoms of depression were more frequent in the index than in the COMP group (54 vs. 36 %, *p* = .032; Table 4).

#### Substance abuse according to the AUDIT and DUDIT

Of the 126 women in the sample, 21 (17 %) reported substance abuse (alcohol and/or drugs). According to the AUDIT, there were no significant differences between groups (14 % in the index vs. 7 % in the COMP group, *p* = .171). According to the DUDIT, there were significantly more drug-related problems in the index group (18 vs. 6 %, *p* = .033; Table 4).

#### Symptoms of hyperactivity and inattention according to the ASRS v. 1.1

Of the 126 women in the sample, 24 (19 %) scored ≥24 on either the inattentive or the hyperactive/impulsive symptom dimension, thus indicating ‘highly probable ADHD’. The women in the index group scored ≥24 significantly more often (31 vs. 14 %, *p* = .019; Table 4).

### Perceived parental stress according to the SPSQ

Both groups of mothers reported significantly greater perceived parental stress than the SPSQ norm (*p* = .0001) (Ostberg 1998). There were no significant differences between the index women (3.23; SD .50) and the COMP women (3.10; SD .61).

## Discussion

The aim of this study was to investigate whether women raised in a family with substance abuse are a particularly vulnerable group of patients in an infant psychiatry setting and to identify the risk factors for suspected parental malfunctioning in women referred to treatment in an infant and toddler intervention program. Treatment in infant psychiatry has the overall goal of facilitating the development of the infant and optimizing the relationship between the child and the parent to help foster a secure child–parent attachment. The development of the infant is in many ways dependent on the parent’s ability to put his or her own needs aside and to focus on the child’s signals. There are many possible different reasons for parents’ inability to do this in an appropriate manner. A commonality among women attending treatment at an infant and toddler intervention program is experiencing stress, anxiety and uncertainty in their parenting (Neander and Engstrom 2009). Many of the women have psychiatric illnesses of their own and experienced complicated childhood conditions that may negatively influence their ability to meet the needs of their infants. During the planning of treatment, it is important to acknowledge the heterogeneity of these mothers, to try to identify relevant background factors and to develop targeted interventions for different groups of patients.

The sample in this study consisted of 126 mothers treated at an infant and toddler unit at a child and adolescent psychiatry clinic in Sweden. The index group of 35 mothers, representing almost a third of the families, reported substance abuse in their family of origin. They were compared with the other mothers, the COMP group (n = 91). The findings suggested that women in the index group were affected more often by factors that could negatively influence their parenting. One of these factors was that more of these women had experienced their own parents’ divorce, and they were more often single mothers. Divorce has a substantial impact on children’s lives, including exposure to interparental conflict and changes in residences and standards of living, and it is a condition that leads to deleterious effects on children’s health-related factors (Weitoft et al. 2003).

Another factor negatively affecting parenting ability was that women in the index group were exposed to different forms of traumatic life events more often. Previous research has suggested that traumatized parents are likely to be less emotionally or functionally available to their children and to have an authoritarian parenting style, including verbal hostility, physical coercion, and low nurturance. Maternal trauma may also be a predictor of the potential for parental abuse (Cohen et al. 2008; Lyons-Ruth et al. 2005; Schwerdtfeger et al. 2013).

A third factor was the higher prevalence of depressive symptoms among the index mothers. Post-partum depression is the most prevalent maternal psychiatric disorder; it occurs in 10–15 % of women and may lead to deficient and insensitive parenting (Reck et al. 2008). Extreme levels of maternal anxiety and depression during pregnancy and the postnatal period have been shown to negatively affect child development and parent–child relationships (Murray et al. 2011; O’Connor et al. 2005; Tronick and Reck 2009). There were no differences between groups in symptoms of anxiety or perceived parental stress. These problems were very frequent among all mothers, far above the Swedish norm, and may, at least partly, have led to their referral for treatment.

A fourth factor was the high proportion of previous substance abuse among the index mothers. One in five of the index women and only one woman in the COMP group reported previous substance abuse. However, these differences in substance abuse were not detected with the AUDIT self-report questionnaire, probably because the instrument assesses only the previous 12 months.

Finally, as hypothesized, symptoms indicative of ADHD were significantly more prevalent in the total sample of patients compared with the prevalence in the general population of adult women (Faraone and Biederman 2005), and ADHD symptoms were even more common in the index group with a family history of substance abuse. According to a recent review, parental ADHD symptoms may be associated with deficits in parenting control behavior, including family disorganization and chaos, less monitoring of child behavior, less effective problem solving in child-rearing, and more inconsistent and over-reactive disciplining (Johnston et al. 2012). Thus, significantly more women in the index group than in the COMP group scored above the cut-off level for ‘highly probable ADHD’ as a ‘proxy’ diagnosis of ADHD. This does not mean that these women had been diagnosed with ADHD. Many women with inattention problems remain undiagnosed until adulthood, when life exerts greater demands on their overall functioning (Quinn and Madhoo 2014). To the best of our knowledge, this is the first report of ADHD symptoms in women treated for parental dysfunction. This is important new knowledge that needs to be considered in the development of treatment models for these patients to account for their difficulties with impulsivity and executive functioning.

Hence, all these factors may separately or in combination have a negative influence on an individual’s ability to practice sensitive parenting. It may also be difficult, in the context of treatment, to identify the specific needs of a person from a substance abusing family due to family dynamics, in which role confusion/parentification is common. Parentified children may perform parenting tasks and act very competent, but they are accustomed to neglecting their own needs (Macfie et al. 2015).

An obvious limitation of the present study is the use of a selected clinical population in which all participants had pronounced difficulties with parental functioning; hence, the findings cannot be applied to all grown children of parents with substance abuse.

Regarding the instruments, the sociodemographic and psychosocial data on the subjects and their family of origin reflect the subjective reports of the respondents. The most severe limitation is the lack of a validated self-report questionnaire for identifying our index group. There is a well-validated instrument, the Children of Alcoholics Screening Test (CAST) (Hodgins and Shimp 1995), that has been used in several larger population studies (Hill et al. 2008); however, because we wanted to use several other validated instruments, and the Regional Ethics Committee recommended a limited number of instruments, we decided to use our own self-report questionnaire instead of the CAST. The HADS has been criticized in the past for not sufficiently distinguishing between anxiety and depression, and it has been suggested that the HADS items should be considered in terms of general distress rather than anxiety and depression specifically (Norton et al. 2013). However, the results have been inconsistent; some studies have lent support to the two-factor model used in this study (Helvik et al. 2011; Norton et al. 2013). This study focused on the role of certain risk factors for insensitive parenting at the expense of others. Furthermore, the cross-sectional design does not allow for conclusions regarding causality.

## Conclusions

In this study of mothers receiving treatment because of impaired parenting behavior, as indicated by their referral to a psychiatry clinic because of these difficulties, a substantial subgroup came from families with substance abuse. The findings suggest that even within a sample treated because of parenting malfunctions, women with substance abuse in their family of origin are a particularly vulnerable group of patients. The findings of a high reported prevalence of traumatic experiences and high levels of ADHD symptoms necessitate special treatment considerations. This knowledge should be important to health professionals, with the implication of screening for anxiety, depression and ADHD before the start of therapy. To prevent the intergenerational transmission of alcohol and drug abuse, it is important to identify parents with pronounced needs and to administer targeted treatment and support at primary health care centers and infant psychiatric clinics. Furthermore, this work suggests that it may be useful for prevention programs to identify at-risk women during pregnancy on the basis of a family history of substance abuse.

## Abbreviations

ADHD: attention deficit hyperactivity disorder
ASRS: Adult ADHD Self-Report Scale
AUDIT: The Alcohol Use Disorder Identification Test
DUDIT: The Drug Use Disorder Identification Test
COMP: the comparison group
HADS: The Hospital Anxiety and Depression Scale
SPSQ: The Swedish Parenthood Stress Questionnaire
SUD: substance use disorder
